# Di­aqua­bis­[2-(2-hy­droxy­eth­yl)pyridine-κ^2^ *N*,*O*]cobalt(II) dichloride

**DOI:** 10.1107/S1600536813018321

**Published:** 2013-07-06

**Authors:** Ouahida Zeghouan, Fatiha Guenifa, Nasreddine Hadjadj, Lamia Bendjeddou, Hocine Merazig

**Affiliations:** a Unité de Recherche Chimie de l’Environnement et Moléculaire Structurale ’CHEMS’, Faculté des Sciences Exactes, Campus Chaabet Ersas, Université Constantine I, 25000 Constantine, Algeria

## Abstract

In the title salt, [Co(C_7_H_9_NO)_2_(H_2_O)_2_]Cl_2_, the Co^II^ cation, located on an inversion center, is *N*,*O*-chelated by two hy­droxy­ethyl­pyridine ligands and coordinated by two water mol­ecules in a distorted O_4_N_2_ octa­hedral geometry. In the crystal, the Cl^−^ anions link with the complex cations *via* O—H⋯Cl hydrogen bonds, forming a three-dimensional supra­molecular architecture. π–π stacking is observed between the pyridine rings of adjacent mol­ecules [centroid–centroid distance = 3.5810 (11) Å].

## Related literature

For applications of pyridine derivatives in the synthesis of coordination polymers, see: Sanudo *et al.* (2003 ▶); Boskovic *et al.* (2002 ▶). For related complexes containing a 2(2-hy­droxy­eth­yl)pyridine ligand, see: Kong *et al.* (2009 ▶); Mobin *et al.* (2010 ▶). For hydrogen-bond motifs, see: Bernstein *et al.* (1995 ▶).
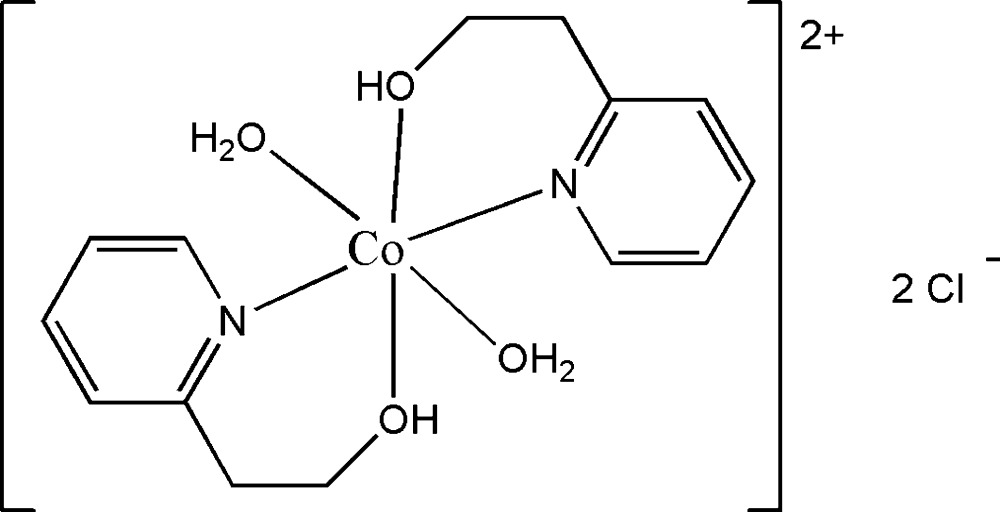


## Experimental

### 

#### Crystal data

[Co(C_7_H_9_NO)_2_(H_2_O)_2_]Cl_2_
*M* *_r_* = 412.17
Orthorhombic, 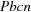

*a* = 12.8911 (3) Å
*b* = 8.0049 (2) Å
*c* = 16.8757 (4) Å
*V* = 1741.44 (7) Å^3^
*Z* = 4
Mo *K*α radiation
μ = 1.31 mm^−1^
*T* = 293 K
0.3 × 0.2 × 0.2 mm

#### Data collection

Bruker APEXII diffractometer
9407 measured reflections
1535 independent reflections
1419 reflections with *I* > 2σ(*I*)
*R*_int_ = 0.015

#### Refinement

*R*[*F* ^2^ > 2σ(*F* ^2^)] = 0.020
*wR*(*F* ^2^) = 0.056
*S* = 1.04
1535 reflections
115 parameters
H atoms treated by a mixture of independent and constrained refinement
Δρ_max_ = 0.21 e Å^−3^
Δρ_min_ = −0.22 e Å^−3^

### 

Data collection: *APEX2* (Bruker, 2006 ▶); cell refinement: *SAINT* (Bruker, 2006 ▶); data reduction: *SAINT*; program(s) used to solve structure: *SIR2002* (Burla *et al.*, 2005 ▶); program(s) used to refine structure: *SHELXL97* (Sheldrick, 2008 ▶); molecular graphics: *ORTEP-3 for Windows* (Farrugia, 2012 ▶); software used to prepare material for publication: *WinGX* (Farrugia, 2012 ▶), *Mercury* (Macrae *et al.*, 2006 ▶) and *POV-RAY* (Persistence of Vision Team, 2004 ▶).

## Supplementary Material

Crystal structure: contains datablock(s) global, I. DOI: 10.1107/S1600536813018321/xu5717sup1.cif

Structure factors: contains datablock(s) I. DOI: 10.1107/S1600536813018321/xu5717Isup2.hkl

Additional supplementary materials:  crystallographic information; 3D view; checkCIF report

## Figures and Tables

**Table 1:** Selected bond lengths (Å)

Co1—O1	2.1210 (13)
Co1—O1*W*	2.0715 (13)
Co1—N1	2.1537 (14)

**Table 2:** Hydrogen-bond geometry (Å, °)

*D*—H⋯*A*	*D*—H	H⋯*A*	*D*⋯*A*	*D*—H⋯*A*
O1—H1⋯Cl^i^	0.84 (2)	2.26 (2)	3.0625 (13)	162 (2)
O1*W*—H1*W*⋯Cl^ii^	0.932 (11)	2.145 (12)	3.0738 (13)	174.6 (19)
O1*W*—H2*W*⋯Cl^iii^	0.835 (16)	2.285 (16)	3.1121 (14)	170.4 (15)

Symmetry codes: (i) 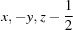; (ii) 
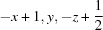
; (iii) 
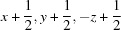
.

**Fig. 1.**
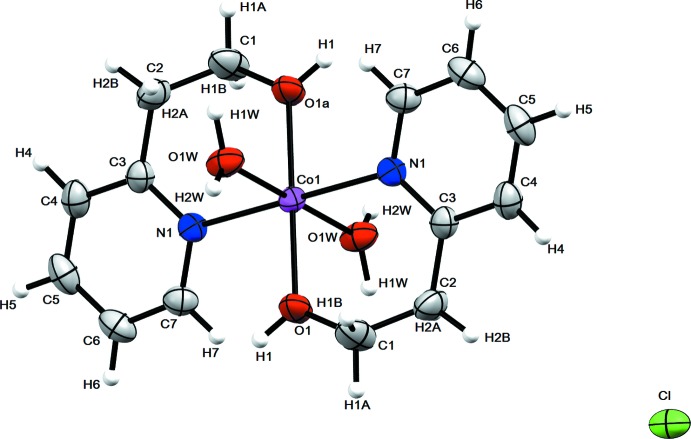
The asymmetric unit of the title structure with the atom numbering scheme. Displacement ellipsoids are drawn at the 50% probability level and H atoms are represented as small spheres of arbitrary radii.

**Fig. 2.**
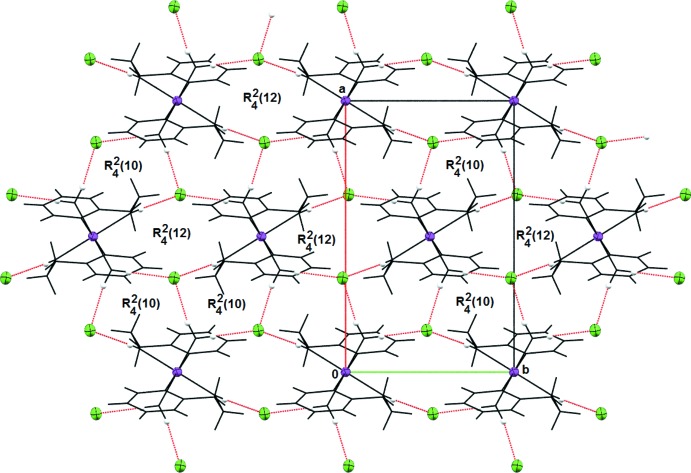
Part of the crystal structure, showing the aggregation of R24(12) and R24(10) hydrogen-bonding motifs.

**Fig. 3.**
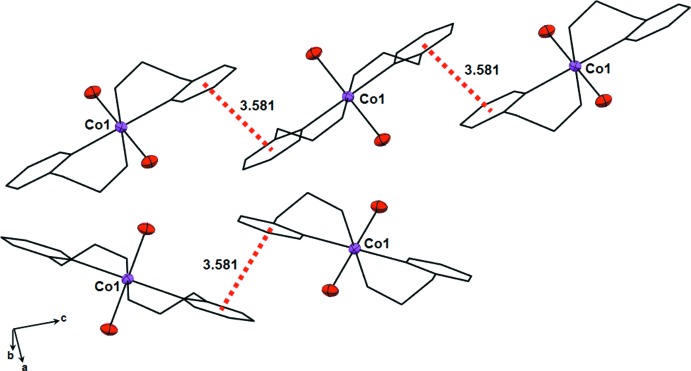
A part of the crystal packing showing π–π stacking interactions between the pyridine rings (dashed lines).

## supplementary crystallographic information

### Comment

Pyridine derivatives ligands have the potential to be used in the synthesis of supramolecular materials, particularly transition metal coordination polymers (Sanudo *et al.*, 2003; Boskovic *et al.*, 2002). A few complexes containing *L* (*L* =2 (2-hydroxyethyl)pyridine) have been studied for years, because this ligand has a versatile coordination activities and bridging function (Kong *et al.*, 2009; Mobin *et al.*, 2010). We report here the synthesis and crystal structure of the title compound.

The complex comprises two *L* (*L* = 2-(2-hydroxyethyl)pyridine) ligands, one Co^II^ ion, two aqua ligands and uncoordinated Cl anions (Fig. 1). The coordination geometry around the Co center is octahedral with a CoN_2_O_4_ ligand set (Table 1). The bis *L* ligands coordinate to the Co(II) ions through the nitrogen atom of pyridine ring and the oxygen atom of hydroxyl group, creating a chelate ring. The octahedral geometries are completed by two *trans* aqua ligands at axial positions.

The complex cations are connected *via* O—H···Cl hydrogen bonds (Fig. 2), forming a centrosymmetric and a noncentrosymmetric rings, in two domensionel network, which can be described by the graph set *R*^2^_4_(12) and *R*^2^_4_(10), respectively (Bernstein *et al.*,1995). π-π stacking between the pyridine rings [centroid-centroid distance = 3.5810 (11) Å] is also present (Fig. 3).

### Experimental

The title compound was prepared by reaction of 2 (2-hydroxyethyl)pyridine (10.0 mmol, 1.67 g) in a mixture of ethanol–water (V/V = 1:1) and CoCl_2_.6H_2_O (10.0 mmol, 2.50 g), the solution was maintained at 313 K under agitation during 24 h at room temperature. Pink crystals were obtained by slow evaporation of the solvents within 3 weeks.

### Refinement

H atoms were placed at calculated positions with C—H = 0.93 Å (aromatic H atoms) and 0.97 Å (methylene H atoms), and refined in ridong mode with U_iso_(H) = 1.2U_eq_(C). The O-bound H-atoms was located in a Fourier map and refined with O—H restraint of 0.85 (1) Å, U_iso_(H) = 1.5U_eq_(O).

### Crystal data

**Table:**

[Co(C_7_H_9_NO)_2_(H_2_O)_2_]Cl_2_	*F*(000) = 852
*M**_r_* = 412.17	*D*_x_ = 1.572 Mg m^−^^3^
Orthorhombic, *P**b**c**n*	Mo *K*α radiation, λ = 0.71073 Å
Hall symbol: -P 2n 2ab	Cell parameters from 1536 reflections
*a* = 12.8911 (3) Å	θ = 3.2–25.1°
*b* = 8.0049 (2) Å	µ = 1.31 mm^−^^1^
*c* = 16.8757 (4) Å	*T* = 293 K
*V* = 1741.44 (7) Å^3^	Prism, pink
*Z* = 4	0.3 × 0.2 × 0.2 mm

### Data collection

**Table:**

Bruker APEXII diffractometer	1419 reflections with *I* > 2σ(*I*)
Radiation source: fine-focus sealed tube	*R*_int_ = 0.015
Graphite monochromator	θ_max_ = 25.1°, θ_min_ = 3.9°
φ scans	*h* = −14→15
9407 measured reflections	*k* = −9→9
1535 independent reflections	*l* = −19→20

### Refinement

**Table:**

Refinement on *F*^2^	0 restraints
Least-squares matrix: full	H atoms treated by a mixture of independent and constrained refinement
*R*[*F*^2^ > 2σ(*F*^2^)] = 0.020	*w* = 1/[σ^2^(*F*_o_^2^) + (0.0293*P*)^2^ + 0.7255*P*] where *P* = (*F*_o_^2^ + 2*F*_c_^2^)/3
*wR*(*F*^2^) = 0.056	(Δ/σ)_max_ < 0.001
*S* = 1.04	Δρ_max_ = 0.21 e Å^−^^3^
1535 reflections	Δρ_min_ = −0.22 e Å^−^^3^
115 parameters	

### Special details

**Table:**

Geometry. Bond distances, angles *etc*. have been calculated using the rounded fractional coordinates. All su's are estimated from the variances of the (full) variance-covariance matrix. The cell e.s.d.'s are taken into account in the estimation of distances, angles and torsion angles
Refinement. Refinement of *F*^2^ against ALL reflections. The weighted *R*-factor *wR* and goodness of fit *S* are based on *F*^2^, conventional *R*-factors *R* are based on *F*, with *F* set to zero for negative *F*^2^. The threshold expression of *F*^2^ > σ(*F*^2^) is used only for calculating *R*-factors(gt) *etc*. and is not relevant to the choice of reflections for refinement. *R*-factors based on *F*^2^ are statistically about twice as large as those based on *F*, and *R*- factors based on ALL data will be even larger.

### Fractional atomic coordinates and isotropic or equivalent isotropic displacement parameters (Å^2^)

**Table:**

	*x*	*y*	*z*	*U*_iso_*/*U*_eq_	
Co1	0.50000	0.50000	0.00000	0.0229 (1)	
O1	0.41357 (11)	0.27513 (16)	0.00777 (7)	0.0360 (4)	
O1W	0.62402 (10)	0.39039 (16)	0.05822 (8)	0.0360 (4)	
N1	0.43197 (11)	0.57142 (18)	0.11153 (8)	0.0272 (4)	
C1	0.35447 (17)	0.2261 (3)	0.07500 (12)	0.0476 (7)	
C2	0.40794 (17)	0.2792 (2)	0.15026 (11)	0.0413 (6)	
C3	0.40051 (14)	0.4625 (2)	0.16765 (10)	0.0288 (5)	
C4	0.36041 (17)	0.5176 (2)	0.23920 (11)	0.0367 (6)	
C5	0.35342 (16)	0.6852 (3)	0.25479 (11)	0.0417 (6)	
C6	0.38502 (15)	0.7962 (2)	0.19758 (12)	0.0404 (6)	
C7	0.42246 (14)	0.7350 (2)	0.12727 (11)	0.0331 (6)	
Cl	0.34635 (4)	0.01817 (5)	0.40317 (3)	0.0402 (2)	
H1	0.3978 (18)	0.211 (3)	−0.0294 (11)	0.0540*	
H1A	0.34570	0.10570	0.07490	0.0570*	
H1B	0.28620	0.27680	0.07240	0.0570*	
H1W	0.6327 (17)	0.2761 (13)	0.0664 (13)	0.0540*	
H2A	0.48060	0.24860	0.14680	0.0500*	
H2B	0.37800	0.21780	0.19420	0.0500*	
H2W	0.6813 (12)	0.436 (2)	0.0664 (14)	0.0540*	
H4	0.33820	0.44050	0.27680	0.0440*	
H5	0.32780	0.72310	0.30310	0.0500*	
H6	0.38110	0.91080	0.20640	0.0490*	
H7	0.44230	0.81090	0.08840	0.0400*	

### Atomic displacement parameters (Å^2^)

**Table:**

	*U*^11^	*U*^22^	*U*^33^	*U*^12^	*U*^13^	*U*^23^
Co1	0.0221 (2)	0.0221 (2)	0.0245 (2)	0.0002 (1)	0.0017 (1)	0.0009 (1)
O1	0.0429 (8)	0.0315 (7)	0.0337 (7)	−0.0128 (6)	0.0066 (6)	−0.0041 (5)
O1W	0.0293 (7)	0.0295 (7)	0.0491 (8)	0.0000 (5)	−0.0083 (6)	0.0071 (6)
N1	0.0259 (7)	0.0276 (7)	0.0280 (7)	0.0020 (6)	0.0025 (6)	−0.0007 (6)
C1	0.0534 (13)	0.0401 (11)	0.0492 (12)	−0.0200 (10)	0.0219 (10)	−0.0088 (9)
C2	0.0564 (13)	0.0310 (10)	0.0365 (10)	−0.0006 (9)	0.0159 (9)	0.0067 (8)
C3	0.0263 (9)	0.0336 (9)	0.0266 (9)	−0.0003 (7)	0.0004 (7)	0.0003 (7)
C4	0.0353 (11)	0.0479 (12)	0.0270 (9)	−0.0029 (8)	0.0034 (8)	−0.0003 (8)
C5	0.0381 (11)	0.0547 (12)	0.0324 (10)	0.0017 (10)	0.0038 (8)	−0.0152 (9)
C6	0.0395 (11)	0.0354 (10)	0.0463 (11)	0.0033 (9)	0.0010 (9)	−0.0134 (9)
C7	0.0306 (10)	0.0297 (9)	0.0389 (10)	0.0004 (7)	0.0022 (8)	−0.0013 (7)
Cl	0.0376 (3)	0.0269 (2)	0.0561 (3)	−0.0017 (2)	−0.0020 (2)	0.0015 (2)

### Geometric parameters (Å, º)

**Table:**

Co1—O1	2.1210 (13)	C2—C3	1.499 (2)
Co1—O1W	2.0715 (13)	C3—C4	1.386 (3)
Co1—N1	2.1537 (14)	C4—C5	1.370 (3)
Co1—O1^i^	2.1210 (13)	C5—C6	1.374 (3)
Co1—O1W^i^	2.0715 (13)	C6—C7	1.371 (3)
Co1—N1^i^	2.1537 (14)	C1—H1A	0.9704
O1—C1	1.422 (2)	C1—H1B	0.9701
O1—H1	0.84 (2)	C2—H2A	0.9699
O1W—H2W	0.835 (16)	C2—H2B	0.9697
O1W—H1W	0.932 (11)	C4—H4	0.9303
N1—C3	1.350 (2)	C5—H5	0.9305
N1—C7	1.342 (2)	C6—H6	0.9307
C1—C2	1.506 (3)	C7—H7	0.9300
			
O1—Co1—O1W	90.95 (5)	N1—C3—C4	121.20 (15)
O1—Co1—N1	87.56 (5)	C2—C3—C4	120.39 (15)
O1—Co1—O1^i^	180.00	N1—C3—C2	118.40 (15)
O1—Co1—O1W^i^	89.05 (5)	C3—C4—C5	120.24 (17)
O1—Co1—N1^i^	92.44 (5)	C4—C5—C6	118.61 (18)
O1W—Co1—N1	90.70 (5)	C5—C6—C7	118.77 (16)
O1^i^—Co1—O1W	89.05 (5)	N1—C7—C6	123.51 (16)
O1W—Co1—O1W^i^	180.00	O1—C1—H1A	109.58
O1W—Co1—N1^i^	89.30 (5)	O1—C1—H1B	109.54
O1^i^—Co1—N1	92.44 (5)	C2—C1—H1A	109.58
O1W^i^—Co1—N1	89.30 (5)	C2—C1—H1B	109.59
N1—Co1—N1^i^	180.00	H1A—C1—H1B	108.04
O1^i^—Co1—O1W^i^	90.95 (5)	C1—C2—H2A	108.66
O1^i^—Co1—N1^i^	87.56 (5)	C1—C2—H2B	108.64
O1W^i^—Co1—N1^i^	90.70 (5)	C3—C2—H2A	108.70
Co1—O1—C1	124.41 (12)	C3—C2—H2B	108.72
C1—O1—H1	107.4 (15)	H2A—C2—H2B	107.60
Co1—O1—H1	127.1 (15)	C3—C4—H4	119.87
H1W—O1W—H2W	107.4 (17)	C5—C4—H4	119.89
Co1—O1W—H2W	125.1 (12)	C4—C5—H5	120.72
Co1—O1W—H1W	125.4 (13)	C6—C5—H5	120.67
C3—N1—C7	117.65 (14)	C5—C6—H6	120.60
Co1—N1—C7	117.99 (11)	C7—C6—H6	120.63
Co1—N1—C3	124.34 (11)	N1—C7—H7	118.22
O1—C1—C2	110.47 (17)	C6—C7—H7	118.27
C1—C2—C3	114.33 (16)		

Symmetry code: (i) −*x*+1, −*y*+1, −*z*.

### Hydrogen-bond geometry (Å, º)

**Table:**

*D*—H···*A*	*D*—H	H···*A*	*D*···*A*	*D*—H···*A*
O1—H1···Cl^ii^	0.84 (2)	2.26 (2)	3.0625 (13)	162 (2)
O1*W*—H1*W*···Cl^iii^	0.932 (11)	2.145 (12)	3.0738 (13)	174.6 (19)
O1*W*—H2*W*···Cl^iv^	0.835 (16)	2.285 (16)	3.1121 (14)	170.4 (15)

Symmetry codes: (ii) *x*, −*y*, *z*−1/2; (iii) −*x*+1, *y*, −*z*+1/2; (iv) *x*+1/2, *y*+1/2, −*z*+1/2.
